# Interventions to reduce relapse risk and drug craving in patients with substance use disorders in forensic psychiatric care: a systematic review of controlled trials

**DOI:** 10.3389/fpsyt.2025.1718332

**Published:** 2025-12-17

**Authors:** Martin Månsson, Peter Andiné, Malin Hildebrand Karlén, Christopher Holmberg

**Affiliations:** 1 Department of Psychiatry for Affective Disorders, Sahlgrenska University Hospital, Region Västra Götaland, Gothenburg, Sweden; 2 Institute of Medicine, Sahlgrenska Academy, University of Gothenburg, Gothenburg, Sweden; 3 Centre for Ethics, Law and Mental Health, Department of Psychiatry and Neurochemistry, Institute of Neuroscience and Physiology, Sahlgrenska Academy, University of Gothenburg, Gothenburg, Sweden; 4 Department of Forensic Psychiatry, Sahlgrenska University Hospital, Region Västra Götaland, Gothenburg, Sweden; 5 Department of Forensic Psychiatry, National Board of Forensic Medicine, Gothenburg, Sweden; 6 Department of Psychology, University of Gothenburg, Gothenburg, Sweden; 7 Department of Psychotic Disorders, Sahlgrenska University Hospital, Region Västra Götaland, Gothenburg, Sweden; 8 Institute of Health and Care Sciences, Sahlgrenska Academy, University of Gothenburg, Gothenburg, Sweden

**Keywords:** forensic psychiatry, randomized controlled trial, controlled trial, systematic review, substance use disorder, forensic mental health

## Abstract

**Systematic review registration:**

Substance use is a risk factor for relapse in violent crime. In forensic psychiatric care (FPC), severe mental disorder and comorbid substance use is common, with a majority having a history of substance use disorder (SUD) and many having committed their index crime while under influence. FPC is dedicated to treating these patients to reduce the risk of criminal recidivism. Though interventions for SUD are used, none of them have been developed to meet the needs of the FPC patient group specifically.

**Aim:**

To identify and evaluate controlled interventions that primarily or secondarily reduce the risk of relapse into substance use and/or feelings of drug craving among FPC patients.

**Methods:**

A systematic review was conducted, spanning 10 years (2014-2024). The PRISMA 2020 guidelines were followed. A PICO framework, and specified inclusion and exclusion criteria, guided the process. In collaboration with an experienced medical librarian a search strategy was developed and searches were conducted in MEDLINE, Embase, and PsycInfo. Blinded screening and article selection through consensus voting was performed collaboratively by two of the authors, as was the full-text reviews. Quality assessment was conducted using the CASP checklist for controlled studies. The results were synthesized using vote counting to determine the direction of effect across studies, following the SWiM reporting criteria.

**Results:**

The searches identified 1275 articles. After deduplication, 750 articles remained. Following screening, 9 articles were assessed in full, of these, 4 were excluded for reporting irrelevant outcomes and 3 for lacking a control group. Finally, 2 controlled trials – both RCTs – were included. Overall quality was adequate with some concerns for bias. No conclusive evidence of a treatment effect on SUD measurements was reported.

**Conclusion:**

This systematic review suggests there is a lack of research aimed at the study of SUD interventions within FPC. Analysis of included articles found no conclusive evidence of effective treatments, but there seems to be an indication of a beneficial effect on drug use in one study, a treatment that addresses impulsivity. Further high-quality studies better tailored to FPC are needed to evaluate SUD treatment outcome among FPC patients.

## 1 Introduction

Forensic psychiatry assesses and treats patients who have been sentenced to forensic psychiatric care (FPC) after a committed crime, a criminal behaviour that has been linked to their severe mental disorder. Common diagnoses of underlying severe mental disorder are – according to ICD-10 (1) – schizophrenia, and other psychotic disorders, neuropsychiatric disabilities, delusional disorders, personality disorders, mood disorders, and intellectual disabilities. Substance use disorder (SUD) is also a common comorbidity and DSM-5 lists 11 diagnostic criteria that are used to determine the severity of the illness (mild, moderate, or severe) (2). Some of the issues reflected in the criteria are social impairment, craving, risky behavior, tolerance, and withdrawal. Common comorbid psychiatric disorders interplaying with SUD, and exacerbating each other, are anxiety, depression and PTSD.

In the FPC population, SUD is a common comorbid diagnosis with ramifications beyond the level of the individual patient. Data from a variety of geographical and cultural backgrounds have shown the close relationship between severe mental illness, substance use, and serious violent and sexual crime in the FPC populations (3, 4). Substance use is an important risk factor for committing and being subjected to crime. The responsibility of the medical profession to promote societal health and improvement is thus especially tangible within a FPC setting, where the psychiatric apparatus must contribute to the mental health of the individual patient while also contributing to crime prevention and public safety.

In Sweden, the RättspsyK Annual report for 2024 shows that the majority of both male and female patients treated within FPC have a history of substance abuse (many having a mixed misuse profile) (5), in line with comparable international figures (3). The same report shows that a large part of the FPC patients were under the influence of narcotics or alcohol at the time of their index crime. Although the large majority of FPC patients with a SUD diagnosis were reported to have received some type of SUD treatment/intervention within FPC, these predominantly consisted of drug screenings, since drug screenings are defined as treatment by the Swedish National Board of Health and Welfare. In comparison, psychoeducative intervention, and pharmacological treatment, towards SUD was considerably less prevalent, leaving an apparent divide between the extent of patients with SUD and the extent of SUD-treated patients. It should also be noted that while drug screening is an intervention in the broad sense of the term, it does not, as the RättspsyK report also points out, necessarily amount to biofeedback for the patient in FPC (by sharing and discussing the results with the patient), but rather serves as a pure control function to safeguard a drug-free environment at that FPC ward. No recommendations are given specifically by Swedish National Care guidelines for the FPC context, but SUD-treatments recommended by national guidelines are both pharmacological treatments, and psychological or psychosocial treatments such as *Cognitive Behavioural Therapy* and *Community Reinforcement Approach.* International research on this co-morbid FPC group has emphasized the importance of simultaneous treatment (6), but to our knowledge, no such treatment structure has been experimentally tested yet.

A 2018 systematic review mapping knowledge gaps in FPC explored five domains internationally within FPC where knowledge was found to be lacking (7). These domains included pharmacological treatments and their effects and side-effects, psychosocial interventions and their outcomes, and psychological interventions and their effects on relapse in substance use. While there are numerous evidence-based treatments for SUD in general, the review found no readily employable interventions designed specifically for the FPC population. Apart from their SUD, these patients suffer from severe mental disorders often with other co-occurring psychiatric and somatic conditions that necessitate polypharmacy and long durations of care. This complexity creates a challenging and resource-intensive patient group, which is crucial to find effective treatments for, to prevent relapse in violent crime.

Further complicating treatment, factors commonly co-occurring with SUD – such as trauma and impulsivity (e.g. through diagnoses such as PTSD or ADHD which are relatively common in FPC populations) – influence patient behavior and affect treatment outcomes (8–11). Beyond the medical complexities, potential treatments face additional hurdles, including low patient motivation under legally mandated conditions (12), the risk of medication misuse or the diversion of medicine from the patient to other persons (13), adherence and insight issues (14), and the societal stigma and legal barriers that can jeopardize successful reintegration (e.g. challenges in securing housing, employment, and rebuilding a supportive social network) (15, 16).

The lack of evidence-based interventions for SUD in the FPC patient groups highlights ethical imperatives outlined in the UN Principles for the Protection of Persons with Mental Illness and the Improvement of Mental Health Care (17). These principles emphasize providing care appropriate to individual needs and the safety of others (Principle 9), and offering the best available mental health care to criminal offenders (Principle 20). Further, the European Convention on Human Rights requires that any person being incarcerated for mental health reasons must receive adequate treatment (18). The complex interplay between severe mental disorder and SUD – especially since SUD is considered such a strong risk factor for relapse and is related to approximately 2.5 years longer involuntary FPC periods for patients – necessitates the quick development of appropriate, scientifically validated interventions. Meeting this need also fulfills ethical obligations to reduce criminal recidivism, benefiting both patients and society. By prioritizing evidence-based treatment approaches for SUD, FPC can minimize reliance on purely punitive measures, which will result in long periods of mere containment, and the administration of unvalidated treatments.

Consequently, there remains a clear need for high-quality research targeting SUD interventions in FPC. Moreover, a synthesis of existing research is essential to pinpoint interventions that are, or could become, beneficial for this complex and heterogeneous patient population.

This review aims to identify interventions, offered to FPC patients in either inpatient or outpatient FPC settings, that primarily or secondarily reduce the risk of relapse into substance use or feelings of drug craving. To answer this, the following objectives were formulated: What interventions reduce the risk of substance-use relapse and/or the severity of drug craving among patients in FPC compared to treatment-as-usual (TAU)? What were the effects of the interventions on the severity of SUD, and the amount of symptoms such as drug craving?

## 2 Material and methods

### 2.1 Study design

To answer the research questions, a systematic review was conducted of primary studies adhering to a pre-specified set of inclusion and exclusion criteria as outlined in **Table 1**, following the PRISMA 2020 guidelines for reporting systematic reviews (19).

**Table 1:** Inclusion and exclusion criteria of research articles.

Inclusion	Exclusion
Randomized controlled trials (RCTs), nonrandomized controlled studies, case-control studies	All other types of studies, e.g. noncontrolled studies, systematic reviews, case studies, case series, editorials etc.
Languages: English, Swedish, Danish, Norwegian	All other languages
Date: January 1 2014 to July 12 2024	All studies pre-2014 and post-July 12 2024
Studies adhering fully to the PICO framework	Studies deviating from the PICO framework

### 2.2 Sampling procedure

The PICO framework (Population, Intervention, Comparison, Outcome) was employed to specify the scope of the database searches, systematize the screening process, and clarify the eligibility for the research articles. The PICO framework is preferred when the intention is to evaluate an intervention’s effect on specific outcomes (20). Further inclusion and exclusion criteria are presented in **Table 1**. This systematic review was based on the findings of an umbrella review published in 2018 (7). The date restriction was, however, set a few years earlier at January 1 2014. This margin was to account for eventual articles that were not yet indexed in 2018. The included research articles met the criteria of PICO according to the following:

#### 2.2.1 Patient/population

FPC patients having reached the age of criminal responsibility (in accordance with the jurisdictions of the research article’s FPC setting), screened for or diagnosed with SUD, and/or suffering from drug craving. Substances included were alcohol, cannabis and illicit drugs including illicit use of medications. Substances excluded were tobacco, and prescribed medications taken according to prescription.

#### 2.2.2 Intervention

Any type of intervention was included, such as pharmacotherapy, psychotherapy, combined therapies, social interventions (e.g. housing, economy etc.).

#### 2.2.3 Comparison/comparator

Studies comparing intervention to nonintervention were deemed highly unlikely to be found due to ethical objections towards non-treatment in FPC. Therefore, the control group consisted of TAU or care-as-usual, in accordance with the needs of the individual research subjects. As such, the intervention group consisted of TAU/care-as-usual with the addition of the intervention in question.

#### 2.2.4 Outcome

The included outcomes were any primary or secondary measurements pertaining to SUD and/or drug craving.

### 2.3 Database searches

#### 2.3.1 Search methods for the identification of research articles

After defining the PICO framework, key words and concepts were identified to serve as the foundation of a search strategy. An experienced medical librarian from the Sahlgrenska University Hospital Library helped to develop the search strategy. Following discussions, they optimized the search strategy vis-à-vis PICO and estimated it to yield 1, 000-1, 500 search results. The final search was performed by the librarian on 12 July 2024 in the databases MEDLINE, Embase, and PsycInfo. The searches combined MeSH terms with free text words using Boolean operators. In total, three search blocks were used, identifying the following research areas: First, forensic psychiatry and forensic mental health. Second, SUD. Third, intervention/treatment. No search terms related to the comparison element in PICO were used since this risked limiting the comprehensiveness of searches. The full search strategy is presented in **Supplementary Table 1**.

#### 2.3.2 Screening and study selection process

**Figure 1:**
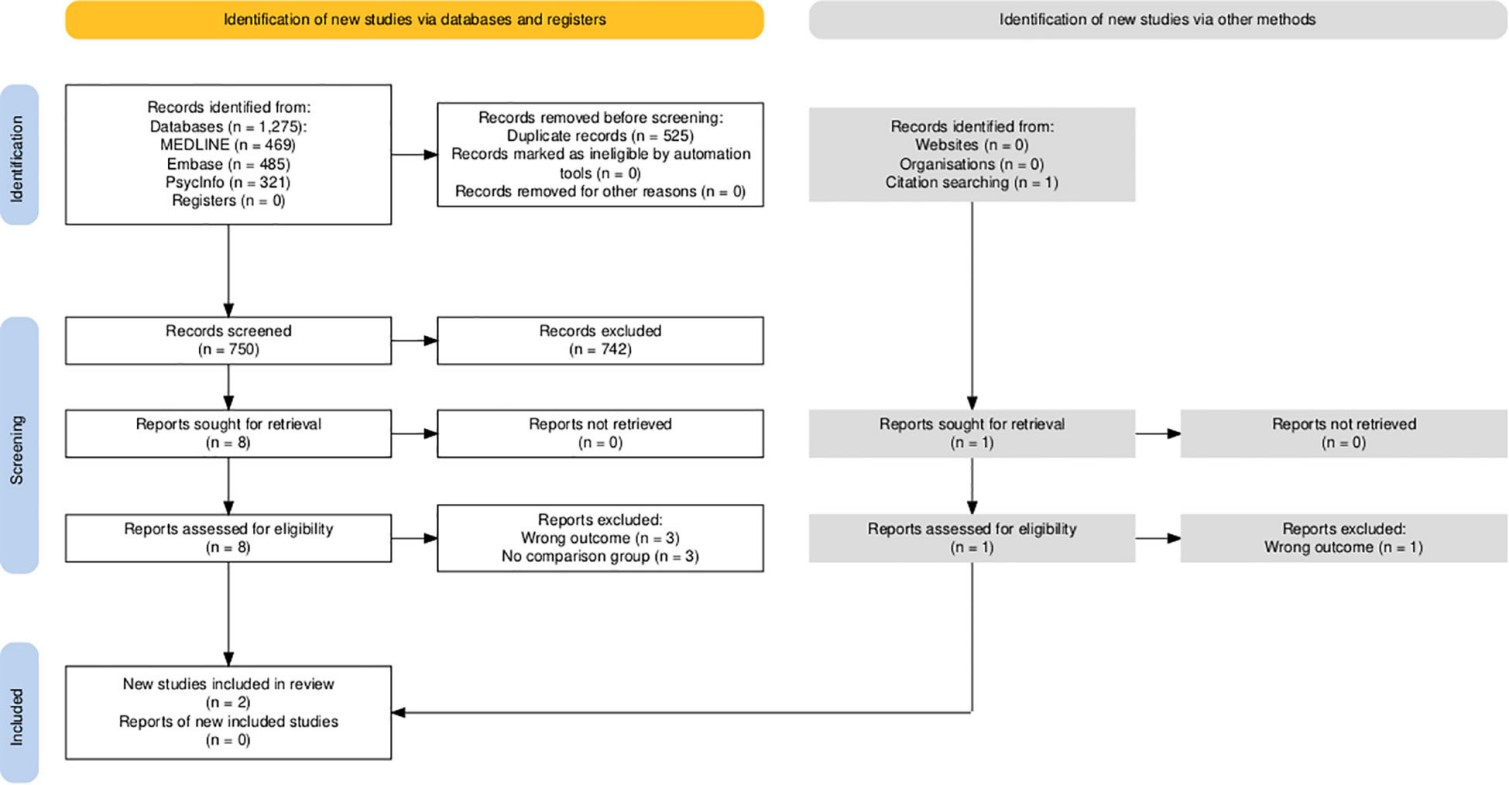
PRISMA2020 flow diagram of the literature search and study selection process.

The study selection process is presented in the form of a PRISMA flow diagram in **Figure 1**, created with the Shiny app (21).

The articles found in the three databases searched were uploaded into Rayyan (https://new.rayyan.ai/) (22). Rayyan is an online screening tool for literature reviews that has been developed to simplify the screening process by blinding users and allowing them to vote individually on the inclusion of research articles. The tool then unblinds the users to facilitate a discussion and an open consensus vote on articles where the voters have been in disagreement. The tool enables two screening methods: the first is a preliminary evaluation of the title, abstract, and metadata, and the second is a comprehensive review of the full text.

Two screeners were used. Would the two screeners disagree, a third person would cast the final vote. A consensus vote was considered to be two screeners voting YES or NO on the same research article. The primary screening was conducted by the authors MM and CH.

The initial database search yielded 1, 275 results across three databases. The librarian removed 520 duplicates, transferring the remaining 755 articles into Rayyan. Five additional duplicates were found using the Rayyan duplicate detection function, leaving 750 articles for screening. After unblinding, 20 research articles received at least one “yes” or “maybe” vote based on the titles and abstracts. These were discussed, and a consensus was reached in all cases without engaging a third screener. This left eight articles for full-text review. A total of 742 articles were excluded during title/abstract screening for failing to adhere to the PICO framework and the inclusion criteria in **Table 1**.

Additionally, 13 systematic reviews from the same time period were identified in the first primary screening that reflected the PICO framework. The 217 references of those 13 systematic reviews were extracted using Zotero and uploaded into a new Rayyan library. Initially, two duplicates were found using the Rayyan duplicate detection function, leaving 215 articles for screening. A total of 214 articles were excluded in the screening of title/abstract for not adhering to the PICO framework and the inclusion criteria in **Table 1**, resulting in one research article further for full text screening. No abstract was found for eight articles, which were subsequently screened on title only.

The secondary full text review was conducted by MM and CH. All nine articles were retrieved from their individual databases. Both screeners read all nine articles, and the contents were discussed and consensus was reached for all articles without engaging the third screener. Of those, four articles were excluded for not presenting a relevant outcome measurement (23–26). Three were excluded for not having a comparison group (27–29), the characteristics of which are presented in **Supplementary Table 2**. Two randomized controlled trials (RCTs) adhered fully to the PICO framework and were included in the systematic review.

All screened articles were written in English.

### 2.4 Quality- and risk of bias-assessments

For the assessment of the research articles included, the CASP Randomised Controlled Trials Checklist was used. CASP or the Critical Appraisal Skills Programme (https://casp-uk.net/) provides checklists for the systematic and critical appraisal of various forms of research papers. For the purposes of randomized controlled trials (RCTs) the checklist contains 13 questions (see **Table 2**) that are intended to identify and summarize that which is “positive/methodologically sound” in the research article, that which is “negative/relatively poor methodology”, and that which is “unknown”, with the objective of helping to assess the quality of the RCT in question in a systematic and repeatable manner across studies (32). The questions have three pre-specified answers, YES, NO, and DON’T KNOW, together with written examples of what to consider when answering.

**Table 2:** Assessment of quality of included articles.

Study	Research question^1^	Participant randomisation^2^	Participant accounting^3^	Participant blinding^4^	Investigator blinding^5^	Assessor blinding^6^	Study group similarity^7^	Level of care^8^	Effects reporting^9^	Precision of estimate^10^	Benefits vs harms & costs^11^	Local applicability^12^	Intervention value^13^
Fielenbach et al., 2018 (30)	Yes	Yes	Yes	No	No	Don’t know	Yes	Yes	Yes	No	Yes	Don’t know	Don’t know
Swinkels et al., 2023 (31)	Yes	Yes	Yes	No	No	Don’t know	Yes	Yes	Yes	Yes	Yes	Don’t know	Don’t know

Quality assessment based on the 13 questions in CASP Checklist: For Randomised Controlled Trials (RCTs), listed as footnotes below. All questions answered with YES, NO, or DON’T KNOW based on the author’s reading of the research articles.

1. Did the study address a clearly formulated research question?

2. Was the assignment of participants to interventions randomised?

3. Were all participants who entered the study accounted for at its conclusion?

4. Were the participants ‘blind’ to the intervention they were given?

5. Were the investigators blind to the intervention they were giving to participants?

6. Were the people assessing/analysing outcome/s ‘blinded’?

7. Were the study groups similar at the start of the randomised controlled trial?

8. Apart from the experimental intervention, did each study group receive the same level of care (that is, were they treated equally)?

9. Were the effects of intervention reported comprehensively?

10. Was the precision of estimate of the intervention or treatment effect reported?

11. Do the benefits of the experimental intervention outweigh the harms and costs?

12. Can the results be applied to your local population/in your context?

13. Would the experimental intervention provide greater value to the people in your care than any of the existing interventions?

**Figure 2:**
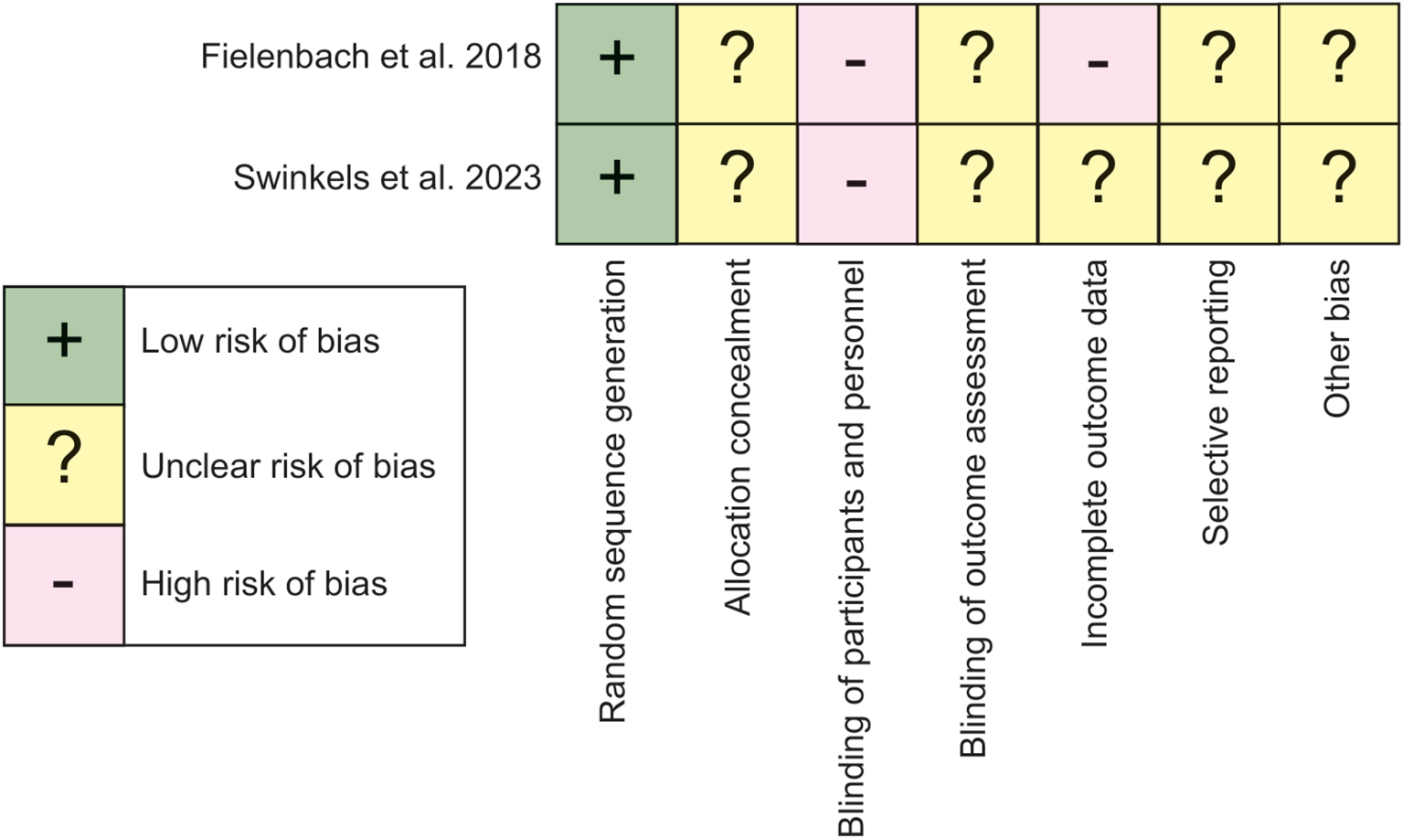
Risk of bias.

A formal risk of bias (RoB) assessment tool was not utilized due to project constraints. However, a principled approach to critical appraisal was undertaken based on the same main principles guiding the Cochrane RoB tool (33). The assessment considered seven key domains of bias: (1) Random sequence generation, (2) Allocation concealment, (3) Blinding of participants and personnel, (4) Blinding of outcome assessment, (5) Incomplete outcome data, (6) Selective reporting, and (7) Other bias. The RoB assessment is outlined in **Figure 2**.

### 2.5 Synthesis of the results of the included articles

The overall high level of heterogeneity in populations and study designs, and further differences in reporting the results and differences in outcome measurements, made conducting a meta-analysis of the collected material inapplicable. The results of the included articles were synthesized through vote counting based on the direction of effect as described in Cochrane Handbook for Systematic Reviews of Interventions (34). Reporting was done broadly following the synthesis without meta-analysis (SWiM) guidelines (35). Though vote counting does not address the difference in size of individual studies or the magnitude of effect, it provides a means to synthesize effect data that differ too greatly for more powerful methods of synthesis. The method divides all results from the different effect measurements into the binary data points of ‘beneficial’ or ‘harmful’, to be able to look at the relative distribution around the null hypothesis of no difference.

Outcome measurements were grouped around a common aspect of SUD such as ‘drug use’ or the specific symptom of ‘drug craving’. To address differences in follow-up time points, only pre- and post-treatment measurements were included in the synthesis to reduce overreporting of studies with in-treatment measurements as well, and to reduce the risk of conflating further any effects of the adaptation to the intervention with the post-treatment effect.

### 2.6 Data collection and calculation

The data were collected manually from the tables and figures in the research articles by MM and confirmed by CH. Mean difference (MD) and 95% CI were calculated to determine the direction of effect of the intervention group treatment relative to control group treatment, for outcome measurements where the relative direction of effect between the groups was not clearly outlined. The calculations were performed by MM and confirmed by CH. The outcome variables relevant for the purpose of this systematic review are outlined in **Table 3**.

**Table 3:** Outcome variables.

Author year country	Study design	Number of patients	Withdrawals – Dropouts	Results^a^		Between-group results		Comments	Directness*	Limitations*	Precision*
				Intervention	Control	MD (95% CI)	RR (95% CI)^b^				
Fielenbach 2018 Netherlands (30)	RCT	*n* = 83	Drug craving *n* = 41 Drug use *n* = 43^c^	Drug craving^d^ Baseline: 44.19 (17.77) 14.1 weeks (5.32): 36.38 (20.45) Drug use^e^ Baseline: 0.53 (0.64) 14.1 weeks (5.32): 0.38 (0.50)	Drug craving^d^ Baseline: 42.72 (17.48) 10 weeks: 39.24 (16.49) Drug use^e^ Baseline: 0.23 (0.31) 10 weeks: 0.22 (0.35)	Drug craving 4.33^f^ (-6.91, 15.57) Drug use 0.14^f^ (-0.13, 0.41)		No patient completed experimental treat- ment within protocol timeframe. No intention-to-treat analysis was performed.	+	?	?
Swinkels 2023 Netherlands (31)	RCT	*n* = 102	12 months *n* = 18 18 months^g^ *n* = 27	Quantity alcohol^h^ Baseline: 9.588 (14.054) 12 months: 7.143 (9.651) 18 months: 5.684 (10.419) Quantity cannabis^h^ Baseline: 0.510 (0.987) 12 months: 0.405 (0.828) 18 months: 0.526 (0.862) Quantity hard drugs^h^ Baseline: 0.216 (0.757) 12 months: 0.238 (0.532) 18 months: 0.105 (0.311) Days alcohol^h^ Baseline: 6.647 (9.492) 12 months: 6.191 (9.253) 18 months: 6.368 (9.604) Days cannabis^h^ Baseline: 9.000 (12.291) 12 months: 8.857 (12.215) 18 months: 10.342 (13.358) Days hard drugs Baseline: 2.078 (5.932) 12 months: 0.810 (3.133) 18 months: 2.237 (6.158)	Quantity alcohol^h^ Baseline: 6.902 (12.911) 12 months: 5.857 (9.527) 18 months: 4.135 (8.011) Quantity cannabis^h^ Baseline: 0.510 (0.834) 12 months: 0.524 (0.917) 18 months: 0.432 (0.765) Quantity hard drugs^h^ Baseline: 0.118 (0.475) 12 months: 0.643 (3.091) 18 months: 0.162 (0.602) Days alcohol^h^ Baseline: 6.137 (9.633) 12 months: 4.310 (7.588) 18 months: 4.028 (7.606) Days cannabis^h^ Baseline: 8.765 (11.911) 12 months: 5.000 (9.571) 18 months: 6.108 (11.007) Days hard drugs^h^ Baseline: 0.412 (1.780) 12 months: 1.071 (3.502) 18 months: 0.405 (1.423)		Quantity alcohol^h^ 1.078 (0.682, 1.702) Quantity cannabis 1.175 (0.726, 1.902) Quantity hard drugs^h^ 0.885 (0.320, 2.451) Days alcohol 0.982 (0.600, 1.608) Days cannabis 1.199 (0.680, 2.112) Days hard drugs 0.693 (0.278, 1.727)	Post-treatment measurements in experimental group included patients that did not complete per protocol intervention in pre-specified 10 months.	?	?	?

* + No or minor problems |? Some problems | – Major problems

Standard deviation (SD) in parenthesis.^a^ Post-treatment average (12 and 18 months).^b^ In both intervention and control groups, *n* = 1 patient data on drug use is missing.^c^ Desire for Alcohol Questionnaire-Short Form (DAQ-SF), modified version.^d^ Historische, Klinische, Toekomst-Revised (Historical, Clinical, Future-Revised).^e^ Showing a larger decrease in favor of intervention.^f^ In outcome measurement Days alcohol, there is *n* = 1 patient data missing in the control group, making a total of *n* = 28 dropouts.^g^ Measurement in the Addictions for Triage and Evaluation 2.1 (MATE 2.1).^h^

RCT, Randomized controlled trial; MD, Mean difference; RR, Rate ratio; CI, Confidence interval.

## 3 Results

### 3.1 Study characteristics

Two RCTs were included in the results. The characteristics of the included articles are presented in **Table 4**.

**Table 4:** Characteristics of included articles.

Author year country	Study design	Length of follow-up	Study groups; intervention vs control	Patients (*n*)	Mean age (years)	Men (%)	Outcome variables
Fielenbach 2018 Netherlands (30)	RCT	10 weeks per protocol 14.1 weeks (SD 5.32) in intervention group	Neurofeedback training + TAU vs TAU only	42*	38.3	100	Drug craving Drug use
Swinkels 2023 Netherlands (31)	RCT	3, 6, and 9 months in-treatment 12 and 18 months post-treatment	Forensic Network Coaching + TAU vs TAU only	102	40.5	88.2	Days of use, and quantities of, alcohol, cannabis and hard drugs

*n = 83 patients were randomized for participation, but only *n* = 42 patients were part of the final per protocol analysis. RCT, Randomized controlled trial; SD, Standard deviation; TAU, Treatment-as-usual.

*The effectiveness of an additive informal social network intervention for forensic psychiatric outpatients: results of a randomized controlled trial* (Swinkels et al., 2023) (31) explored the effects of an additive informal social network intervention called Forensic Network Coaching (FNC) together with TAU, as compared to only TAU, for a FPC outpatient sample in a Dutch context. The purpose of FNC is to provide forensic outpatients with positive social influence through matching and having regular meetings with an informal peer-level coach, aiming at increasing patient well-being and reducing criminal recidivism and hospitalization. Secondary outcome measurements included days [of use] and quantities of alcohol, cannabis and illicit drugs.

*Effects of a Theta/Sensorimotor Rhythm Neurofeedback Training Protocol on Measures of Impulsivity, Drug Craving, and Substance Abuse in Forensic Psychiatric Patients With Substance Abuse: Randomized Controlled Trial* (Fielenbach et al., 2018) (30) explored the effects of a theta/sensimotor rythm neurofeedback training protocol together with TAU, compared to only TAU, on a FPC all-male inpatient population in a Dutch context. The Neurofeedback training is used with the aim of reducing impulsivity with the rationale of measuring levels of drug use and drug craving that impulsivity is intrinsically associated with the initiation and maintenance of SUD.

#### 3.1.1 Study participants

In the Swinkels (2023) study *n* = 102 participants were randomized, and in the Fielenbach (2018) study *n* = 83 participants were randomized, leaving a total of *n* = 185 participants across studies. The Fielenbach (2018) study did however not include an intention-to-treat analysis, including only *n* = 42 patients in the final per protocol-analysis.

#### 3.1.2 Study interventions

In the Swinkels (2023) study, *n* = 51 patients were allocated to receive FNC+TAU. In the Fielenbach (2018) study, *n* = 42 patients were allocated to receive neurofeedback training+TAU. No limitations were posed on TAU. *Treatment supervisors* in the Fielenbach (2018) study were asked to avoid adjusting medical regimens for the duration of the trial.

#### 3.1.3 Study comparison groups

Both studies report that the comparison group received TAU: In the Swinkels (2023) study, *n* = 51 patients were allocated to TAU. TAU comprised forensic care (CBT and/or forensic flexible assertive community treatment).

In the Fielenbach (2018) study, *n* = 41 patients were allocated to TAU. No limitations were posed on TAU. *Treatment supervisors* in the Fielenbach (2018) study were asked to avoid making adjustments to medical regimens for the duration of the trial where possible.

#### 3.1.4 Study outcomes

In the Fielenbach (2018) study, all applicable outcome measurements were primary, namely overall drug craving and drug use. However, in the Swinkels (2023) study, relevant outcome measurements were all secondary and consisted of number of days of use and total quantities of alcohol, cannabis, and *hard drugs* separately. Although the authors did not explicitly operationalize the term ‘hard drugs’, their usage appears consistent with the Dutch legal framework. According to the Dutch Ministry of Justice and Security, a categorical distinction between ‘hard drugs’ and ‘soft drugs’ is formally instituted. Soft drugs are defined as substances with comparatively low hazardousness to health, such as cannabis, hypnotics, and sedatives. Hard drugs, by contrast, encompass substances associated with substantially greater risk of addiction, psychiatric comorbidity, and physiological harm, such as heroin, amphetamine, cocaine, LSD, and MDMA (ecstasy). Different instruments and follow-up time points were used between the studies:

##### 3.1.4.1 Measurement instruments

Swinkels (2023) used self-reported data on number of days and total quantities of different substance use according to the *Measurement in the Addictions for Triage and Evaluation 2.1* (MATE 2.1) (36).

Fielenbach (2018) used self-reported data from a modified version of the *Desire for Alcohol Questionnaire-Short Form* (DAQ-SF) (37) to account for drug craving in general and not only alcohol. Scoring was calculated on 14 items on an ordinal Likert-scale from 1 to 7 (1=‘strongly disagree’ and 7=‘strongly agree’). Drug use was measured using the risk assessment scale *Historische, Klinische, Toekomst-Revised* (Historical, Clinical, Future-Revised) (38), scores ranging from 0 to 4 on the item “substance abuse” where 0 was “no drug use whatsoever” and 4 “the patient tested positive at least twice and refused to undergo drug testing”. Points were scored on positive urine or breathalyzer test for any drug, and for refusal to undergo drug testing.

##### 3.1.4.2 Follow-up time points

The Swinkels (2023) study had in-treatment follow-up at 3, 6, and 9 months. Post-treatment follow-up was at 12 and 18 months. Due to protocol deviations, post-treatment follow-up at 12 and 18 months included patients that had not completed the per protocol minimum of 10 months of FNC-coaching.

Per protocol the Fielenbach (2018) study intervention should have lasted 10 weeks, with follow-up post-treatment at 10 weeks. Mean length of intervention completion and follow-up was due to protocol deviations (planning issues and patient issues), however, 14.1 weeks (SD 5.32).

### 3.2 Vote counting of the direction of effect

**Figure 3:**
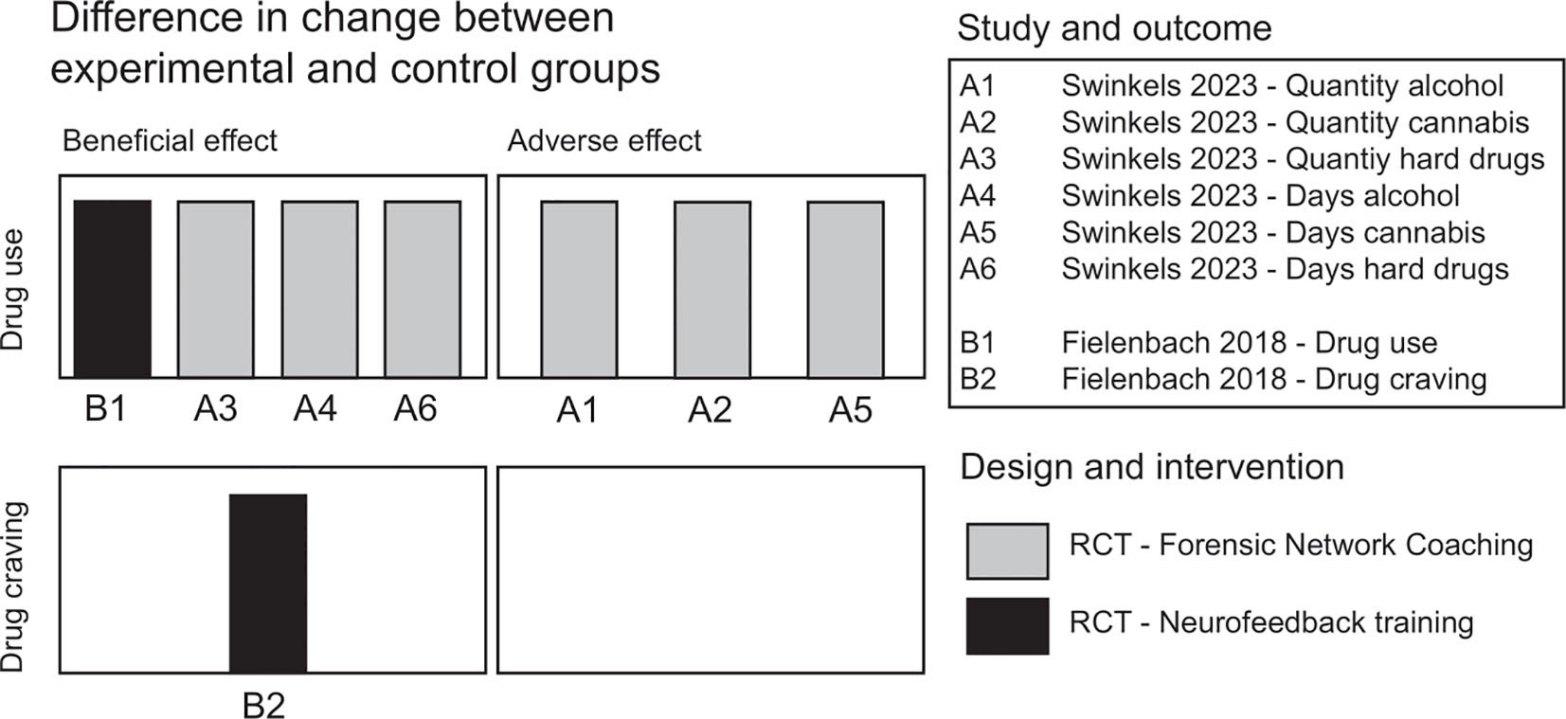
Result of vote counting of direction of effect. Summary of the evidence of effect on drug use and drug craving between the included studies. Each bar is of the same height and represents one vote as to the direction of the effect (i.e. beneficial effect or adverse effect) according to study and outcome. Study design and intervention is depicted using different colors of the bars.

The between-group outcome variables for the vote counting of the direction of effect are outlined in **Table 3**. The results of the vote counting are presented in **Figure 3**.

Fielenbach (2018) reported pre-treatment measurements, and single post-treatment measurements on the outcome measurements of ‘drug craving’ and ‘drug use’, thus rendering two outcome categories for vote counting. The relative direction of effect between the intervention group and the control group was not clearly outlined, therefore MD and 95% CI were calculated for the purpose of vote counting and assessing the validity of the measurements.

Swinkels (2023) reported pre-treatment measurements, and in- and post-treatment measurements at multiple time-points. Six parallel measurements were used in the outcome category of ‘drug use’; the outcome measurements used were *days of use*, and *quantities*, of *alcohol*, *cannabis*, and *hard drugs* separately. No single-best outcome measurement could thus be selected to represent the outcome category as a whole. Out of a total of 29 outcome measurements across all time points, the average post-treatment effect – as provided by the study – was chosen for each of the six parallel measurements. By choosing six outcome measurements for the vote counting, overreporting was reduced to the minimum possible while focusing on post-treatment effects across both studies.

### 3.3 Quality of the included research articles

The quality of the included research articles was assessed using the CASP checklist for RCTs containing 13 questions to help in the evaluation (32). The questions and answers are presented in **Table 2**. Quality across both studies was deemed as satisfactory when comparing positive, negative, and unknown methodological factors, with some concerns of bias. Firstly, both studies had overall positive characteristics in terms of randomized study design and comprehensive reporting, adding to the validity of the findings. Secondly, both studies had some methodological problems with blinding and deviations from the study protocols. The nature of the interventions given where the blinding of whether you were having an informal social contact person or not was unfeasible in the Swinkels (2023) study, as was whether you were conducting neurofeedback training as in the Fielenbach (2018) study. With neurofeedback training you nevertheless do have the possibility to perform sham-neurofeedback training, as acknowledged by the article authors. This was, however, not utilized in this specific study for the control group, considered a limitation by the authors. Further, none of the studies accounted for whether the person(s) analyzing the outcomes were “blinded” to the data collected, introducing some possibility of bias skewing the results. Both studies acknowledged unforeseen deviations from the study protocols in terms of length of intervention at the time of post-treatment follow up due to compliance issues in Fielenbach (2018), and problems with coach-matching in Swinkels (2023). Thirdly, the benefits of both studies seem to outweigh the harm looking at the overall outcome measurements (i.e. taking into account effect measurements that do not directly pertain to SUD), with no study showing any serious adverse effects pertaining to the experimental treatments given. These benefits are however less clear when only looking at the outcome measurements pertaining to SUD in the Swinkels (2023) study. Lastly, the inconclusiveness of the results, and cultural and jurisdictional differences, puts into question whether or not the results could add any value outside a Dutch context. Both studies were performed in the Netherlands and while there are similarities in baseline characteristics between the country’s FPC populations, potential issues exist where Dutch legislation allows for persons with severe personality disorders to be committed to FPC. Similarly, cultural expressions of normative drug and alcohol use differ in some areas, with the Netherlands having legalized personal use of cannabis, whereas in several other countries (including Sweden) it remains illegal.

### 3.4 Risk of bias

The assessment of the risk of bias of the research articles is presented in **Figure 2**.

#### 3.4.1 Randomization, allocation concealment, and blinding

Both studies used random sequence generation, Fielenbach (2018) through a random number generator, and Swinkels (2023) an online randomization tool (Castor EDC), reducing the risk of selection bias. None of the studies report any measures of concealing the allocation, even though it seems to exist as a feature in Castor EDC. Blinding was not possible in any of the studies, due to the overt nature of the interventions. No information concerning the blinding of outcome assessments was given in either study.

#### 3.4.2 Incomplete outcome data

Fielenbach (2018) reported deviations from the study protocol where no patient in the intervention group was able to complete the neurofeedback within the pre-specified 10 weeks, instead mean time to completion was 14.1 weeks (SD 5.32). Further, some patient data (*n* = 2) was missing in the drug use analysis, and the authors do not specify any statistical measures employed to account for this. No intention-to-treat analysis was performed. The analyses performed included patients that had undergone pre- and post-treatment measurements, which resulted in excluding half of the randomized patients from the final analysis, and hence affected the concern of bias. The dropout proportion was the same in both groups, and while the study acknowledged attrition, there was only limited comparison of possible reasons for this between the intervention group and the control group.

Swinkels (2023), similarly, suffered problems with dropout and compliance, with several patients not having been able to complete the pre-specified 10 months of FNC within the given timeframe of 12 months due to difficulties with matching patients to coaches. Further, patients not showing up for follow-up appointments were reported. Dropout was, however, similar across groups, and statistical methods were employed to address missing outcome data. The in-treatment measurements had, furthermore, some evident problems with two instances of nonsensical results in the self-reported data, adding some concern of bias. An intention-to-treat analysis was included.

#### 3.4.3 Selective reporting

Both studies were preregistered and reported the intended outcome variables. The reporting was transparent and comprehensive across studies, with all participants that entered the studies accounted for. Fielenbach (2018) did, however, not report any CIs, only *p*-values for all outcome measurements. Swinkels (2023), in parallel, introduced some exploratory *post hoc* analyses, reporting all CIs but no exact *p*-values. Cutoffs at *p* < 0.05, *p* < 0.01, and *p* < 0.001 were, however, provided.

#### 3.4.4 Other bias

Both studies used self-reported data. In both studies, however, measures were employed to reduce eventual bias in patient self-reported data. Fielenbach (2018) used urine and breathalyzer tests to measure and monitor patient drug use. However, the refusal to provide a urine sample or perform a breathalyzer test was scored as a positive drug test. As such, the scoring used must be viewed as in part possibly misleading as there was no objective way to know if a patient refusing to cooperate would have tested positive. Further, the scale used by Fielenbach (2018) to measure drug craving (DAQ-SF (37)) was developed for alcohol craving, but modified here to account for all drug craving, possibly affecting the sensitivity of the tool.

The inherent diversity in the FPC populations in terms of underlying psychiatric disorders, and for the purpose of this systematic review what kind of SUD is present, introduces the risk of the subgroups responding differently to the intervention making it harder to draw conclusions about possible effects of the intervention. Especially when paired together with the risk of imbalance between intervention and control groups due to the relatively small sizes of the trials. As TAU is tailored to the specific needs of the individual patient and thus is variable, this also runs the risk of being a further inherent source of confounding bias across both in-study groups and the studies per se, with no apparent possibility of determining at what rate non-protocol interventions were balanced between the groups studied in either of the RCTs. This together with the relatively small sizes of the trials leaves an increased risk of imbalances in baseline characteristics between intervention and control groups, and between the studies per se, that could skew the results. The most salient example of imbalances in baseline characteristics in these studies is possibly the fivefold difference between women in the intervention group and the control group in the Swinkels (2023) study (19.6% vs 3.9%), which could add to the confounding bias. Gender role stereotypes and other gender-specific influences, which are not accounted for in the evaluation of the interventions, may contribute to differences in treatment, in addition to lower exposure to SUD among women. A further point is that specific TAU medications such as aripiprazole can have a dampening effect on the level of drug craving experienced by the patients, as noted by Fielenbach (2018).

### 3.5 Adverse effects

No adverse effects specific to the intervention were reported in either study. Swinkels (2023) could, conversely, show a significant reduction in hospitalization and criminal behaviour in the intervention group. Fielenbach (2018) reports aggressive incidents in some patients affecting the timeline of the experimental intervention, but those aggressive incidents seem to stem from the patients’ underlying psychiatric problems and not the intervention per se. Swinkels (2023) reports one patient randomized to the FNC-intervention no longer being eligible pre-treatment due to a personal crisis, another patient in the intervention-group died before the 18-month assessment from unrelated causes.

### 3.6 Heterogeneity

There are several sources of heterogeneity both within, and between, the individual studies that complicate the interpretation of the results and the applicability of the interventions in forensic psychiatry as a whole: Inherent heterogeneity in groups of FPC patients is the wide range of underlying mental disorders, inherent group-level differences between the sexes, different substance(s)-of-choice vis-à-vis SUD, and differences in TAU where according to Swinkels (2023) certain medications can have effect on the data collected such as clozapine on EEG, and others on outcome measures such as the dampening effect of aripiprazole on drug craving (39). The relatively small size of the studies in this systematic review adds to the risk of selection bias between the intervention and control groups due to the inherent heterogeneity in baseline characteristics, complicated further by different levels of compliance as reported in both studies. Between-group heterogeneity exists in the form of differences in patient and study characteristics such as gender division and level of care where Swinkels (2023) studied a mixed population of male and female patients in an outpatient setting, whereas Fielenbach (2018) studied an all-male inpatient population. For the purpose of this review, the inclusion of both inpatient and outpatient settings were chosen since there were few studies in this field and no strong indication on whether a certain type of patient setting would have RCTs or not. The availability of illicit drugs should be different between an inpatient and an outpatient setting, and similarly, the severity level of the psychiatric symptomatology would differ depending on whether the patient is in their inpatient or outpatient phase of their FPC. Further differences arise: First in the study designs where Swinkels (2023) studied FNC as a means of positive reintegration into society, and Fielenbach (2018) studied neurofeedback training as a means of reducing impulsivity. Second in outcome measurements where the two studies looked at drug use with different scales and different levels of subdivision, and Fielenbach (2018) alone looked at drug craving as well.

### 3.7 Evaluation of the evidence of the research papers’ results

The evaluation of study directness, limitations, and precision, towards the outcome measurements of interest for this review are presented in **Table 3**.

Fielenbach (2018) had both drug use and drug craving as primary outcome measurements, presenting a clear theoretical foundation for linking impulsivity to SUD, giving it a high degree of directness. The study was, however, limited by protocol deviations, few participants, and the experimental status of the intervention given, besides the general concerns arising from heterogeneity and the risk of bias. There were, furthermore, some issues with precision as no 95% CI was presented. Precise *p*-values were, however, provided.

Swinkels (2023) had secondary outcome measurements pertaining to drug use. The primary outcome measurement ‘mental wellbeing’, as well as key secondary outcome measurements ‘general psychiatric functioning’, ‘hospitalization’, and ‘criminal recidivism’, are associated with drug use, but any direct link to an effect on actual drug use is less clear. This study also had limitations in terms of protocol deviations, few participants, problems with self-reported data, and again general concerns arising from heterogeneity and associated risks of bias. Concerning precision, 95% CI was provided. However, no exact *p*-values were given, presenting only cutoffs at *p* < 0.05, *p* < 0.01, and *p* < 0.001.

## 4 Discussion

This systematic review synthesizes the results of two RCTs regarding the outcome categories ‘drug craving’ and ‘drug use’ in FPC patients. No conclusive evidence of an effect was found in either category, and the vote counting according to direction of effect gives no strong indication of a beneficial (or detrimental) effect across studies. The interventions and populations studied do, however, have substantial differences which render comparisons difficult. One of the largest problems is perhaps the comparison of an inpatient group in Fielenbach (2018) to an outpatient group in Swinkels (2023). Further, Swinkels (2023) uses FNC, a treatment method seeking positive change through the patient’s social context. Fielenbach (2018), on the other hand, approaches the problems of impulsivity and SUD through neurofeedback training, at the biological level of eliciting change.

### 4.1 Drug use

Fielenbach (2018) studied overall drug use, hypothetically, but not specifically, excluding alcohol consumption, while Swinkels (2023) studied days, and quantities, of alcohol, cannabis and hard drugs separately. Vote counting of post-treatment results showed no obvious overall direction of effect. Four outcome measurements were beneficial, and three were harmful, and none reached statistical significance. However, six of those outcome figures represented average post-treatment measurements from the Swinkels (2023) study, thus making this study overrepresented in the vote counting, potentially biasing the balance of results. Nevertheless, these six outcome measurements showed a mixed trend being evenly distributed around the null hypothesis of no difference, whereas Fielenbach (2018) indicated a beneficial effect on overall drug use. Although the Swinkels (2023) study is thus overrepresented, this remains a deliberately cautious interpretation, as the pattern shows neither a clearly beneficial nor harmful trend. Including additional post-treatment measurements at 12 and 18 months from the Swinkels (2023) study would shift the vote counting to nine favourable versus four unfavourable votes, suggesting a slight overall beneficial trend also in that study. This would, however, further influence the balance of results between studies, with more unclear validity. Therefore, the description of the findings as “mixed” should be understood as a conservative summary of a highly heterogenous evidence base rather than an inference of equivalence or null effect.

Interestingly, in the Swinkels (2023) study, the mean scores on days of hard drug use and quantity of hard drugs were both beneficial, while the cannabis measurements were both detrimental, and alcohol was balanced around the null hypothesis of no difference. As both studies were conducted in the Netherlands, where cannabis is legal for personal use, a decrease in hard drug use coupled with an increase in cannabis use may partly reflect a broader cultural context of acceptance of cannabis use in different social settings. Research has shown that cannabis users generally have more positive attitudes towards legalization (40), and that Dutch adolescents use cannabis more than the European average, influenced by social norms and attitudes (41). This may to some extent explain the lack of positive evaluation in this category.

Fielenbach (2018) showed a decreasing trend of drug use in the neurofeedback group as compared to the control group (*p* = 0.10). Though not statistically significant, it represents the strongest finding of a beneficial effect across studies. Interestingly, this trend is present even though no patient was able to learn the whole neurofeedback protocol, reiterating the need of determining the proper length and intensity of neurofeedback training in this patient group to better ascertain the certainty of any beneficial effect. It is also important to note that the purpose of neurofeedback of effecting impulsivity only carries an indirect relationship to SUD. Impulsivity is proxy to SUD as well as other factors that in turn could influence drug use and drug craving. SUD behaviour is therefore not directly related to impulsivity as such.

Further studies with a design to address the problems with heterogeneity and sources of bias are needed to determine which FPC patients could benefit from which interventions to lower their levels of drug and alcohol use. Increased sample sizes are needed to be able to make subgroup analyses for different psychiatric diagnoses. To broaden the knowledge of how the treatments could affect levels of use of drugs in light of the possible ambiguity stemming from the different jurisdictions and attitudes towards drugs such as cannabis, further studies are needed across different cultural and jurisdictional contexts.

As FPC patients are a heterogeneous group with regards to baseline characteristics such as psychiatric disorder(s), any research done carries the risk of “diluting” treatment effects. Though some of this risk could be mitigated by larger samples, SUD carries further heterogeneity with different levels of severity, different drug(s) of choice, and different comorbidities potentially moderating treatment access and how the patients benefit. It could be that RCTs, however rigorous in design, entails a kind of “one suit fits all” approach that is not as aligned to the variability of forensic psychiatric TAU and comorbidity as with other study populations, especially with the added aspect of variability in SUD. Further considerations regarding proper study designs to ascertain what treatment regarding SUD works within a FPC context are, however, beyond the scope of this review.

Other constraints in regards to the complexity of comorbid disorders is the lack of integrated care, and the existence of structural limitations in FPC are explored in a recent interview study (42). Examples mentioned are the unequal status of SUD care across FPC units, and the risk of staff attitudes to penalizing substance use relapse instead of viewing it more as a normal step on the road to recovery (and work with that) (43). The importance of creating a therapeutic alliance is highlighted in interview studies with both FPC patients and staff (42, 43), but several challenges for the individual such as stigma, distrust of authorities, low psychosocial functioning, and a certain mismatch between staff perceptions and their own needs as described by the patients, exists that complicates the feasibility of effectively treating SUD, and would need to be overcome both in clinical and experimental settings.

### 4.2 Drug craving

Drug craving was addressed by Fielenbach (2018) alone, but the between-group effect observed was minimal with no discernable significance (*p* = 0.89). Interestingly, time as a factor for change had instead a significant beneficial effect across both the intervention and the control groups (*p* = 0.02), which might not be all that surprising as this was an inpatient sample. Time x group-analysis might be slightly in favor of intervention (*p* = 0.34).

Further studies with a better study design, that incorporates intention-to-treat analysis and larger samples, could possibly ascertain whether subpopulations of FPC patients could lower levels of drug craving faster with neurofeedback training. The authors also acknowledge the uncertainty regarding proper length and intensity of neurofeedback training, adding another area of research needed to advance treatment applicability. Further, studies should investigate the effects of medication on the EEG spectrum more closely before applying neurofeedback or should at least control for medication intake during the analysis to achieve more conclusive results. A study from 2020 has shown a general limitation of knowledge of pharmacological treatment in the context of FPC (44). Another limitation concerning medication is the fact that some medications, such as aripiprazole, are known to have beneficial effects on levels of craving (39) which could have influenced the results on the craving questionnaire DAQ-SF.

### 4.3 Comparison to earlier research on substance use disorder treatment in forensic psychiatric care

#### 4.3.1 Absence of high-quality research

In line with the results of this review, several recent articles indicate that there is a general lack of high-quality controlled studies, and cost-benefit analyses, in FPC research, limiting methodological strength across the study of multiple types of interventions (44–48). Many studies carry issues such as small sample sizes and a lack of control groups, increasing risk of bias and limiting the generalizability of the results (45, 46, 48). As an example, looking at pharmacological treatment, comorbid conditions and participant selection criteria are often not transparently reported, leading to difficulties in assessing drug efficacy (44). Similar issues arise in psychological interventions where differences in outcome measurements and study methodologies makes it difficult to compare results across patient populations (46, 48).

#### 4.3.2 Heterogeneity in forensic psychiatric care populations and intervention types

As identified in this review, problems to study patients in FPC with more rigorous designs were apparent in the study articles. The issue of heterogeneity of psychiatric disorder, comorbidity, type/severity of SUD, and other factors, such as criminal history and patterns of violent behaviour, are important factors that also have been brought up in several earlier research articles (44–49). Research efforts can have complications, as one intervention may have varying effects depending on the specific condition of the patient and whether they are in an inpatient or outpatient period of FPC. As such, the setting also varies, with interventions being delivered in forensic hospitals, or in the community (as FPC outpatients), each of which having their own security, legal, and therapeutic issues (45, 46, 48).

#### 4.3.3 Substance use disorder – understudied and poorly addressed

SUD is widespread in FPC. This is, however, often understudied or treated as a secondary concern in FPC research (6, 46, 48). Despite there being a strong correlation between SUD and criminal recidivism, many studies focus on the reduction of violence or the improvement of psychiatric symptoms without addressing SUD directly. Often when SUD is included in research, the type of substance and what effect it has on intervention outcomes is rarely explored. Based on the available evidence, it is possible to argue that an integrated approach, delivering treatment both for the “main” psychiatric disorder and SUD within *the same* treatment contact (e.g. psychotherapy, psychoeducation), could improve patient outcomes (6). Further, certain interventions – such as cognitive behaviour therapy, or substance use treatment programme – could have the potential to improve SUD and associated issues such as criminal behavior, but the evidence base remains weak due to methodological problems and small sample sizes (45, 46, 48). Another example is arts therapies in FPC that may help patients with self-regulation, but no conclusion of effectiveness could be drawn from a 2023 meta-analysis due to a lack of high-quality studies (45).

#### 4.3.4 Outcome measurements and long-term follow-up

A limiting factor in this review was the lack of standardized outcome measurements, making it difficult to synthesize the study results. This was also brought up as a problem by several earlier studies, together with the lack of long-term follow-up (44, 46, 48). Few experimental studies seem to track issues such as SUD-relapse, criminal recidivism, and psychiatric stability over a longer time (year(s)), limiting understanding of long-term treatment benefits and risk of SUD-relapse (45, 46, 48). Additionally, studies often use self-reported data that has, as indicated by this review, an increased risk of bias and can be manipulated (48). To increase the reliability of the data, clinician-rated psychiatric assessments and more objective measurements such as toxicology screens should be employed.

#### 4.3.5 Economic aspects and cost-effectiveness

One of the criteria in evaluating the two included RCTs was the applicability in a local Swedish context. As such, a health-economic analysis could be valuable. This kind of analysis was, however, not included in the individual studies. Economic evaluations of FPC interventions seem to be few, making it difficult to determine the cost-effectiveness and financial sustainability of the treatments (47). One aspect brought up in earlier research is the possibility of reducing long-term costs by reducing criminal recidivism through therapeutic communities, comparable perhaps to FNC in the Swinkels (2023) study, or mental health courts, but robust evidence is lacking (49). One major issue is FPC, public health, and the criminal justice system operating on separate budgets, making economic assessments across agencies difficult (47).

### 4.4 Strengths and limitations

To our knowledge, this is the first systematic review of controlled trials on SUD treatment within FPC.

Strengths include a PICO-based eligibility framework (**Table 1**), adherence to PRISMA 2020, Cochrane, and SWiM guidance, and study appraisal using the CASP RCT checklist, with risk-of-bias judgments informed by Cochrane principles (**Figure 2**) (33–35). An experienced medical librarian optimized a comprehensive search across major databases using MeSH and free text terms, including Spanish-language terms (**Supplementary Table 1**); no eligible Spanish-language studies were identified.

Key limitations are methodological and field-level. First, the UK term “compulsory setting” was not included, which may have led to missed records; future reviews should broaden jurisdiction-specific terminology. Second, several studies—one included—did not clearly distinguish participants with versus without SUD; although SUD is highly prevalent in FPC, clearer case definitions are needed. Third, substantial heterogeneity in populations, interventions, and outcomes precluded meta-analysis. We therefore synthesized findings via vote counting of direction of effect, a low-power approach that ignores effect magnitude; to limit over-representation, we reported the smallest feasible set of post-treatment outcomes, consistent with Cochrane and SWiM guidance (34, 35). More robust evidence will require larger samples, harmonized outcome measures, and clearer SUD definitions to enable meta-analysis and strengthen inference. We also wish to raise the absence of pre-registration of the study protocol as a limitation.

## 5 Conclusion and implications

This systematic review of almost 1, 000 screened abstracts identified two controlled studies in the form of RCTs which evaluated the effects of experimental treatments in FPC on measurements of SUD. This indicates that there has been some progress as compared to an earlier comprehensive umbrella review published in 2018 (7) that could not find any high-quality research on interventions targeting SUD in FPC. The included studies did, however, not present any conclusive evidence of effective treatments for the two outcomes, SUD in general, or drug craving specifically. In one of the studies addressing impulsivity in FPC (30), there was, however, some indication of a beneficial effect regarding overall drug use in the intervention group, but the study contained several methodological issues that impacted the validity of the results. In the other study addressing a social network intervention in FPC (31), there was some indication of a beneficial effect on illicit drug use (apart from cannabis), but the effect on overall drug use was less clear. Further, when considering the results of these two studies together, there was no meaningful indication of any overall harmful or beneficial effect between studies. A larger material with more comparable measurement data would be needed to perform a meta-analysis. While these are ambitious initiatives to conduct controlled interventional trials within these complex settings with this heterogeneous patient population, more research is still needed on SUD in FPC since the evidence-base is too weak.

Concerning possible paths for future research, using larger sample sizes would be warranted to mitigate sources of heterogeneity and to be able to introduce subpopulation analyses. Further, there is a need to develop the tools/methods used (to increase measurement sensitivity), and to ascertain the effects of certain medications such as aripiprazole in FPC on the measurements made. These comments are made with the acknowledgment of the limitations posed on the researchers by difficulties in organizing and motivating a substantial FPC patient population to take part in RCTs offered. Complex issues with psychiatric comorbidities, together with current limitations in FPC, begs the question as to what degree it is possible to evaluate treatment effect using standard RCT methodology in this heterogeneous patient population.

## Data Availability

The original contributions presented in the study are included in the article/**Supplementary Material**. Further inquiries can be directed to the corresponding authors.
