# Correction: Interventions to reduce relapse risk and drug craving in patients with substance use disorders in forensic psychiatric care: a systematic review of controlled trials

**DOI:** 10.3389/fpsyt.2026.1809029

**Published:** 2026-02-20

**Authors:** Frontiers Production Office

**Affiliations:** Frontiers Media SA, Lausanne, Switzerland

**Keywords:** forensic psychiatry, randomized controlled trial, controlled trial, systematic review, substance use disorder, forensic mental health

The affiliations were erroneously written for all authors as:

Martin Månsson^1,2^*, Peter Andiné ^2,3,4^, Malin Hildebrand Karlén^1,2,3^ and Christopher Holmberg^5,6^*

^1^Centre for Ethics, Law and Mental Health, Department of Psychiatry and Neurochemistry, Institute of Neuroscience and Physiology, Sahlgrenska Academy, University of Gothenburg, Gothenburg, Sweden

^2^Department of Forensic Psychiatry, Sahlgrenska University Hospital, Region Västra Götaland, Gothenburg, Sweden

^3^Department of Psychology, University of Gothenburg, Gothenburg, Sweden

^4^Department of Forensic Psychiatry, National Board of Forensic Medicine, Gothenburg, Sweden

^5^Department of Psychotic Disorders, Sahlgrenska University Hospital, Region Västra Götaland, Gothenburg, Sweden

^6^Institute of Health and Care Sciences, Sahlgrenska Academy, University of Gothenburg, Gothenburg, Sweden

The correct affiliations for all authors are:

Martin Månsson^1,2^*, Peter Andiné^3,4,5^ Malin Hildebrand Karlén^3,5,6^ and Christopher Holmberg^7,8^*

^1^Department of Psychiatry for Affective Disorders, Sahlgrenska University Hospital, Region Västra Götaland, Gothenburg, Sweden

^2^Institute of Medicine, Sahlgrenska Academy, University of Gothenburg, Gothenburg, Sweden

^3^Centre for Ethics, Law and Mental Health, Department of Psychiatry and Neurochemistry, Institute of Neuroscience and Physiology, Sahlgrenska Academy, University of Gothenburg, Gothenburg, Sweden

^4^Department of Forensic Psychiatry, Sahlgrenska University Hospital, Region Västra Götaland, Gothenburg, Sweden

^5^Department of Forensic Psychiatry, National Board of Forensic Medicine, Gothenburg, Sweden

^6^Department of Psychology, University of Gothenburg, Gothenburg, Sweden

^7^Department of Psychotic Disorders, Sahlgrenska University Hospital, Region Västra Götaland, Gothenburg, Sweden

^8^Institute of Health and Care Sciences, Sahlgrenska Academy, University of Gothenburg, Gothenburg, Sweden

The original version of this article has been updated.

