# Supplementary material for: Interventions to reduce relapse risk and drug craving in patients with substance use disorders in forensic psychiatric care: a systematic review of controlled trials
Source: Front Psychiatry. 2025 Dec 17;16:1718332. doi: 10.3389/fpsyt.2025.1718332 (PMC12753910; doi:10.3389/fpsyt.2025.1718332)
Supplement: Supplementary file 1 [file DataSheet1.pdf]

Supplementary Table 1 - Search strategy.

Database: Ovid MEDLINE(R) ALL (OvidSP)
Date: 2024-07-12
No of results: 469 records

#	Searches	Results
1	exp Substance-Related Disorders/ or exp Narcotics/ or exp Illicit Drugs/ or exp Designer Drugs/	436715
2	(substance* or (drug adj3 abuse*) or (drug adj3 misuse) or (drug adj3 dependen*) or (drug adj3 "use") or (illicit adj3 drug*) or cannabis or addiction or addicted or alcohol or alcoholism or amphetamine* or cocaine* or narcotic* or heroin* or opioid or opioids or marijuana or withdrawal).ab,kf,ti.	1068108
3	1 or 2	1229627
4	exp Forensic Psychiatry/	41059
5	(forensic and (psychiatry or psychiatric or mentally ill or mentally disordered or mental)).ab,kf,ti.	7313
6	4 or 5	44892
7	(treatment* or therapy or therapies or rehabilitat* or program* or model or intervention*).ab,kf,ti.	10992306
8	3 and 6 and 7	1450
9	limit 8 to (danish or english or norwegian or spanish or swedish)	1298
10	limit 9 to yr="2014 -Current"	469

exp/ = term from the Medline controlled vocabulary, including terms found below this term in the MeSH hierarchy
adj1 / adj3 = next to each other, in any order, up to 0 / 2 word(s) in between
.ab,kf,ti. = abstract, author keyword and title
***** = truncation of word for alternate endings

Database: Embase 1974 to 2024 July 10 (OvidSP)
Date: 2024-07-12
No of results: 485 records

#	Searches	Results
1	exp drug dependence/ or illicit drug/ or narcotic agent/ or designer drug/ or exp drug abuse/ or "substance use"/ or "cannabis use"/	435511
2	(substance* or (drug adj3 abuse*) or (drug adj3 misuse) or (drug adj3 dependen*) or (drug adj3 "use") or (illicit adj3 drug*) or cannabis or addiction or addicted or alcohol or alcoholism or amphetamine* or cocaine* or narcotic* or heroin* or opioid or opioids or marijuana or withdrawal).ab,kf,ti.	1394709
3	1 or 2	1524471
4	exp forensic psychiatry/	13764
5	(forensic and (psychiatry or psychiatric or mentally ill or mentally disordered or mental)).ab,kf,ti.	11077
6	4 or 5	19699

7	exp drug dependence treatment/	26910
8	(treatment* or therapy or therapies or rehabilitat* or program* or model or intervention*).ab,kf,ti.	14438505
9	7 or 8	14443526
10	3 and 6 and 9	1300
11	limit 10 to (embase or medline)	1101
12	limit 11 to (danish or english or norwegian or swedish)	928
13	limit 12 to yr="2014 -Current"	485

exp/ = term from the Embase controlled vocabulary, including terms found below this term in the Emtree hierarchy
/ = term from the Embase controlled vocabulary, does not include terms found below this term in the Emtree hierarchy
adj1 / adj3 = next to each other, in any order, up to 0 / 2 word(s) in between
.ab,kf,ti. = abstract, author keyword and title
***** = truncation of word for alternate endings

Database: **APA PsycInfo via EBSCOhost Research Databases**
Date: 2024-07-12
No of results: 321 records

#	Query	Results
S12	Limiters - Publication Date: 20140101-20241231<br><br>Narrow by Language: - danish<br>Narrow by Language: - english	321
S11	S3 AND S6 AND S9<br><br><br>Narrow by Language: - danish<br>Narrow by Language: - english	800
S10	S3 AND S6 AND S9	920
S9	S7 OR S8	2,118,510
S8	TI ((treatment* or therapy or therapies or rehabilitat* or program* or model or intervention*)) OR AB ((treatment* or therapy or therapies or rehabilitat* or program* or model or intervention*))	2,115,175
S7	DE "Addiction Treatment" OR DE "Detoxification" OR DE "Medication-Assisted Treatment" OR DE "Substance Use Treatment" OR DE "Substance Use Treatment" OR DE "Alcohol Treatment" OR DE "Drug Courts" OR DE "Protective Behavioral Strategies" OR DE "Twelve Step Programs"	35,557
S6	S4 OR S5	14,496

S5	TI ((forensic and (psychiatry or psychiatric or mentally ill or mentally disordered or mental))) OR AB ((forensic and (psychiatry or psychiatric or mentally ill or mentally disordered or mental)))	10,397
S4	DE "Forensic Psychiatry"	8,690
S3	S1 OR S2	376,723
S2	TI ((substance* or (drug N4 abuse*) or (drug N4 misuse) or (drug N4 dependen*) or (drug N4 use) or (illicit N4 drug*) or cannabis or addiction or addicted or alcohol or alcoholism or amphetamine* or cocaine* or narcotic* or heroin* or opioid or opioids or marijuana or withdrawal)) OR AB ((substance* or (drug N4 abuse*) or (drug N4 misuse) or (drug N4 dependen*) or (drug N4 use) or (illicit N4 drug*) or cannabis or addiction or addicted or alcohol or alcoholism or amphetamine* or cocaine* or narcotic* or heroin* or opioid or opioids or marijuana or withdrawal))	360,081
S1	DE "Addiction" OR DE "Drug Addiction" OR DE "Substance Use Disorder" OR DE "Alcohol Use Disorder" OR DE "Cannabis Use Disorder" OR DE "Drug Abuse" OR DE "Drug Dependency" OR DE "Inhalant Abuse" OR DE "Opioid Use Disorder" OR DE "Tobacco Use Disorder" OR DE "Substance Related and Addictive Disorders" OR DE "Addiction" OR DE "Nonsubstance Related Addictions" OR DE "Substance Use Disorder" OR DE "Alcohol Use Disorder" OR DE "Alcohol Abuse" OR DE "Alcohol Intoxication" OR DE "Alcohol Withdrawal" OR DE "Drug Abuse" OR DE "Polydrug Abuse" OR DE "Inhalant Abuse" OR DE "Glue Sniffing" OR DE "Opioid Use Disorder" OR DE "Heroin Use Disorder" OR DE "Morphine Dependence" OR DE "Narcotic Drugs" OR DE "Narcotic Agonists" OR DE "Narcotic Antagonists" OR DE "Opiates" OR DE "Opioid Analgesics" OR DE "Narcotic Agonists" OR DE "Pentazocine" OR DE "Tramadol" OR DE "Narcotic Antagonists" OR DE "Nalorphine" OR DE "Naloxone" OR DE "Naltrexone" OR DE "Opiates" OR DE "Codeine" OR DE "Endogenous Opiates" OR DE "Heroin" OR DE "Morphine" OR DE "Papaverine" OR DE "Designer Drugs"	146,932

N0 / N3 = Next to each other, in any order, up to 0 / 3 word(s) in between

TI = title

AB = abstract

DE = subject heading from APA Thesaurus of Psychological Index Terms

***** = truncation of word for alternate endings
