# Supplementary material for: Interventions to reduce relapse risk and drug craving in patients with substance use disorders in forensic psychiatric care: a systematic review of controlled trials
Source: Front Psychiatry. 2025 Dec 17;16:1718332. doi: 10.3389/fpsyt.2025.1718332 (PMC12753910; doi:10.3389/fpsyt.2025.1718332)
Supplement: Supplementary file 2 [file DataSheet2.pdf]

Article reference	Aim and/or objectives	Overall design	Population / sample characteristics	Intervention and how the intervention is measured	Detailed list of outcomes and how outcomes are measured	Outcome results and how they are measured
Marshall, Kletzka, Kanitz, Opperman & Rockwell (2024). Effectiveness of Dialectical Behavior Therapy in a Forensic Psychiatric Hospital. Journal of the American Academy of Psychiatry and the Law, 52:196–206.	Evaluate DBT's effectiveness in reducing symptoms and ward incidents and improving skills/knowledge among forensic inpatients.	Quasi-experimental pre–post observational evaluation; patient-reported measures at baseline and ~6 months; behavioral outcomes across four treatment quarters; no randomization.	Adult inpatients at a maximum-security forensic hospital (2010–2023), predominantly male; majority NGRI/IST legal status; large clinical cohort.	Standalone DBT skills groups or comprehensive DBT; repeated skills modules. Measurement via validated scales and electronic medical record (EMR) tallies.	Self-report: AAQ-II (psychological inflexibility), PHQ-9 (depression), BSL-23 (borderline symptom severity), DBT-WCCL (skills use & dysfunctional coping). Knowledge: DBT curriculum quizzes. Behavioral: assaults, behavioral dyscontrol incidents, and PRN psychotropic use (EMR counts).	Significant pre→post decreases on AAQ-II, PHQ-9, BSL-23; DBT knowledge increased (notably emotion-regulation & interpersonal-effectiveness modules); across four quarters, assaults and PRN administrations decreased; DBT skills-use changes mixed/NS.
Arsuffi & Scarborough (2023). A service evaluation of the Behavioural Treatment for Substance Abuse (BTSA) programme for forensic dual diagnosis populations. J Forensic Psychol Res Pract, 23(2):201–226.	Evaluate whether BTSA improves motivation/readiness to change, locus of control, confidence to abstain, and general self-efficacy; explore participant experiences qualitatively.	Mixed-methods service evaluation; repeated measures (pre, post, ~2-month follow-up) plus post-program focus group.	Adult males with serious mental disorder and coexisting substance misuse in low-secure/rehab wards or community forensic services in two UK areas; N=38 across six cohorts (focus group n=4).	BTSA delivered twice weekly for 35 sessions by NHS staff + Recovery Champions; components: motivational interviewing, contingency management (attendance-based payments), goal setting, social skills, psychoeducation, relapse prevention. Measured with standardized psychometrics across timepoints.	SOCRATES (Recognition, Ambivalence, Taking Steps; drug/alcohol forms); Drug-Taking Confidence Questionnaire (DTCQ-8); IPC Locus of Control (Internal, Powerful Others, Chance); General Self-Efficacy Scale (GSES). Self-report at pre, post, and follow-up.	Repeated-measures ANOVA showed a significant increase in DTCQ-8 from pre→post (attenuated at follow-up but > baseline); SOCRATES subscales moved in expected directions with mixed statistical significance; qualitative themes highlighted validation, psychoeducation, and recognizing progress.
Reiners, Opitz-Welke, Konrad & Voulgaris (2022). Availability of opioid agonist treatment and critical incidents in Forensic Clinics for Dependency Diseases in Germany. Frontiers in Psychiatry, 13:961549.	Describe national availability and clinical practice of opioid agonist treatment (OAT) in FCDD and quantify critical incidents (violence, relapse, escape, reoffending) and discharge modes in 2018.	Cross-sectional observational survey of all German FCDD via anonymous postal questionnaire (2018); descriptive statistics and OAT vs non-OAT comparisons (Fisher's exact, t-test, Wilcoxon).	15/46 clinics (33%) responded; 2,483 patients treated in 2018; 18% relocated to prison due to treatment termination, 15% discharged successfully. Among 7 clinics offering OAT, 1,153 patients treated and 99 (8.6%) received OAT (3.9% of all 2,483).	OAT availability/practice (medications and policies) self-reported by clinics: buprenorphine/naloxone, buprenorphine, methadone, levomethadone; morphine in one clinic; diamorphine none. Reasons for starting/ending OAT and monitoring (e.g., urine samples, diversion) captured via questionnaire.	Clinic-reported counts in 2018: critical incidents (violent behavior against patients/staff, escape during relaxation of security measures, escape from clinic, offense during relaxation), discharge mode (successful vs terminated/relocated), number receiving OAT, and reasons for OAT termination (illegal drug/opioid use, refusal of urine samples, diversion).	Total critical incidents=275: violence vs patients 103 (37.5%), vs staff 39 (14.2%), escape during relaxation 118 (42.9%), escape from clinic 3 (1.1%), offense during relaxation 12 (4.4%). No differences between OAT vs non-OAT clinics on critical incidents or successful discharge; clinics with OAT had higher treatment termination rates relative to total treatments ($p<.007$).
